# The effect of vitamin c supplementation on intercellular adhesion molecule-1 (ICAM-1) concentration on male adolescent obesity in Padang

**DOI:** 10.1186/1687-9856-2013-S1-P86

**Published:** 2013-10-03

**Authors:** Hernofialdi, Eka Agustia Rini, Rizanda Machmud

**Affiliations:** 1 Endocrinology Division, Pediatric Health Departement of Medical Faculty Andalas University, Dr. M. Djamil Hospital Padang

**Keywords:** Adolescent, Obesity, Vitamin C, ICAM-1

## Background

The role of Vitamin C as antioxidant has been known. Obesity is an oxidative stress condition which becomes risk factor of cardiovascular disease, which could be detected through ICAM-1 concentration.

## Objective

To examine the effect of Vitamin C supplementation on the ICAM-1 concentration on male adolescent obesity in Padang.

## Method

The randomized double blind controlled trial of 40 obese adolescent boys aged 14-18 years old were performed on March-May 2011. Subjects were classified into 2 groups, the Vitamin C 500 mg treatment group and the control group which got placebo, twice daily for 8 weeks. The ICAM-1 concentration of both groups was examined before and after the treatment. The data were analyzed with paired sample t-test and independant t-test with significant degree p<0.05.

## Result

The mean of initial ICAM-1 concentration in Vitamin C and placebo group were 370.37 (SD155.09) ng/ml and 232.96 (SD 106.48) ng/ml, respectively. After 8 weeks treatment, the mean of ICAM-1 concentration on the vitamin C and placebo group were 183.32 (SD 52.34) ng/ml and 185.06 (SD 52.34) ng/ml; the mean of ICAM-1 concentration decreased in both group with p 0.007 and <0.05, concecutively. The reducing Delta of ICAM-1 concentration in vitamin C and placebo group were 187.04 (SD 131.5) ng/ml and 47.91 (SD 62.25) ng/ml, respectively. The reducing Delta of ICAM-1 concentration in Vitamin C group were higher than placebo group, with ratio 3.9 : 1 (p<0.05).

## Conclusion

Vitamin C supplementation in obese adolescent boys reduce the ICAM-1 concentration.

